# Development and Evaluation of the Chronic Time Pressure Inventory

**DOI:** 10.3389/fpsyg.2019.02717

**Published:** 2019-12-04

**Authors:** Andrew Denovan, Neil Dagnall

**Affiliations:** Department of Psychology, Manchester Metropolitan University, Manchester, United Kingdom

**Keywords:** chronic time pressure, Chronic Time Pressure Inventory, confirmatory factor analysis, invariance testing, perceived stress

## Abstract

The negative effects of chronic time pressure (i.e., time shortage and feelings of being rushed) are pervasive within modern society. Noting this, and the absence of an established self-report measure, the present paper developed and evaluated the Chronic Time Pressure Inventory (CTPI). Established theory informed the generation of items, resulting in an initial 15-item measure. Study 1, using parallel analysis, exploratory factor analysis and confirmatory factor analysis, examined CTPI factorial structure within a sample of 401 respondents. Additionally, reliability (omega and alpha) and convergent validity testing occurred by correlating the CTPI with the Perceived Stress Scale (PSS-10). Study 2 replicated the emergent, superior factor model in an independent sample of 163 respondents and assessed measurement invariance. Analysis further examined reliability (omega and alpha) and convergent validity. Across the two studies, results supported a bifactor solution, where a general overarching factor encompassed two discrete, but overlapping temporal factors (i.e., Feeling Harried and Cognitive Awareness of Time Shortage). Invariance testing indicated invariance of form, factor loadings, item intercepts and residuals across Study 1 and 2. The CTPI also demonstrated good internal reliability and satisfactory convergent validity with the PSS-10. Findings supported Szollos’ (2009) theoretical conceptualization of chronic time pressure and established the CTPI as a psychometrically sound, theoretically aligned measure of the construct. Indeed, results advocate the CTPI as a promising instrument for conducting survey-based research into chronic time pressure.

## Introduction

### Background to Time Pressure

Within modern society, perceived shortage of time is a frequently experienced aspect of daily life (Goode, 1960; Levine, 1997; Zuzanek, 1998). Recognizing this, a range of academic disciplines have investigated time pressure. This breadth of interest has resulted in a variety of conceptualizations (Szollos, 2009). These include (time): pressure (Teuchmann et al., 1999), stress (Csikszentmihalyi, 1997), crunch (Zuzanek, 2004), famine (Robinson and Godbey, 1999), poverty (De Graaf, 2003), deficit (Bianchi et al., 2005), squeeze (Jacobs and Gerson, 2004), sickness (Dossey, 1982), and scarcity (Hirsch, 1976). Examination of these terms reveals they possess a similar underlying meaning. Accordingly, researchers often use these labels interchangeably (Robinson and Godbey, 1999). In this context, at a general level, subjective ‘time pressure’ refers to the notion that there is insufficient time available to complete necessary tasks (perceived acute time shortage) (Roxburgh, 2004; Kleiner, 2014).

Concomitant with the notion of ‘pressure’ is the concept of temporal overload. This denotes the busy, hastening pace of life, the need to multitask, and the necessity to perform tasks faster. Indicative terms include tyranny of the moment (Eriksen, 2001), fast time (Eriksen, 2001), 24-h society (Kreitzman and Sassone-Corsi, 1999), pace of life (Garhammer, 2002), hyperculture (Bertman, 1998), and work intensification (Menzies and Newson, 2007) (see Szollos, 2009). At a phenomenological level, subjective time pressure comprises experience of both tempo (accelerated pacing of time) and limits/choices (having to select one action over another) (Dapkus, 1985).

At a higher conceptual level, consideration of the perceived time pressure literature reveals a focus on global temporal concerns or consequences (Southerton, 2003; Southerton and Tomlinson, 2005). Particularly, feeling harried and pressed for time. Harried signifies the need to adhere to myriad social practices within precise timeframes, and ‘pressed for time’ designates a general absence of free time (Southerton and Tomlinson, 2005). Thus, harried embodies the negative sense of feeling rushed to a point where time concerns produce worry and anxiety (Brannen, 2005).

Relatedly, Nowotny (1994) introduced the term ‘extended present’ to describe hastened moment-by-moment daily living. This encompasses perceptions of speeded up time, fragmentation resulting from multitasking, and the awareness that constant interruptions frustrate task completion. These acuities result in constant feelings of busyness, unnoticed passing of time, and a lack of opportunity to plan prospectively.

Furthermore, Robinson and Godbey (1999) employ the concept of time deepening to explain how people under temporal constraints work more intensely. Time deepening involves adopting strategies, such as tight scheduling, speeding up activities, multitasking and selecting faster (leisure) activities. Time pressure is also conceptualized in other related ways. For instance, Southerton (2003) observed that people create ‘hot spots’ for work, which free up time for ‘cold spots’ of slow time allotted to family and leisure. The limitations of ‘hot spots’ are that they can create mental strain, a sense of being rushed, and require disciplined adherence.

### Negative and Positive Consequences of Time Pressure

There is strong evidence to suggest that experience of persistent time pressure is associated with poorer health and reduced quality of life (e.g., lower health and life satisfaction) (Gunthorpe and Lyons, 2004). This is especially true when individuals are unable to mentally detach or “switch off” from work (Sonnentag and Bayer, 2005). Indeed, Zuzanek (1998) reported a negative correlation between extreme levels of time pressure and mental health. This relationship may arise from the fact that acute time pressure can act as a physical and psychological stressor, attendant with a range of undesirable outcomes (i.e., inability to cope, sleeping difficulties, tension and fatigue).

Although investigations of chronic time pressure typically report that sustained perceived temporal constraints negatively affect health and psychological wellbeing, there exists a substantial body of work that has demonstrated that time pressure can facilitate positive social behavior (see review by Capraro, 2019). Illustratively, in a series of experiments using economic games, Rand et al. (2012) found that participants who reached decisions faster were more cooperative. Other researchers have produced similar outcomes (see Rand, 2016). However, studies also report null results (Verkoeijen and Bouwmeester, 2014; Bouwmeester et al., 2017).

Researchers have also produced commensurate positive results for altruism (Rand et al., 2016), honesty (Shalvi et al., 2012; Capraro, 2017; Lohse et al., 2018; Capraro et al., 2019), and the equity-efficiency trade-off (Capraro et al., 2017). In the case of altruism, it is important to note that Rand et al. (2016) only observed a positive effect for women (there was no significant effect for men).

Collectively, investigations demonstrate that perceived time pressure or shortage can have both positive and negative consequences depending on duration, context and individual perceptions (Rudd, 2019). This reflects the subjective perception of time pressure. Particularly the fact that individuals can view it either as motivating, or as something to endure (Lallement and Gourmelen, 2018).

### Conceptualization of Chronic Time Pressure (Szollos, 2009)

Noting that lack of consensus and heterogeneity of terminology hindered the conceptual development of time pressure, Szollos (2009) identified core elements that define the construct. These embrace the notion that subjective experience of time shortage is negative (i.e., aversive, unwanted, undesirable, and apprehension inducing) and coalesce around two factors, time shortage (perceived lack of time), and being rushed (sense of time passing quickly). The latter two experiences overlap, but represent discrete factors.

Time shortage implies objective problems with time allocation, involves cognitive based judgment, and produces minimal affect. Hence, the term is often synonymous with time-management. Contrastingly, feeling rushed focuses on subjective emotional experiences, and is experiential in nature. Accordingly, feeling rushed typically gives rise to feelings of apprehension, worry, anxiety and frustration.

Noting this distinction, in an attempt to establish theoretical clarity, Szollos (2009, p. 339) defined time pressure as, “a temporary, overarching designation that would subsume all the terms related to time shortage as well as to being rushed.” This delineation acknowledged cognitive awareness of insufficient time and the emotional experience of hastened pace. Furthermore, Szollos (2009) definition linked time pressure with stress-related research. Explicitly, it recognized that perceived time pressure was transactional, arising from the interaction between the individual and their environment.

An additional conceptual advantage of linking time pressure with stress is that the association emphasizes the chronic consequences that time shortage can have on the individual. The word ‘chronic’ in this circumstance is important because it delimits time pressure as a habitual, reoccurring, repetitive, and potentially aversive process. Noting these features, Szollos (2009) advocated adoption of the nomenclature chronic time pressure (CTP).

Theoretically, CTP provides a solid platform for empirical inquiry. Explicitly, it facilitates the investigation of time pressure at a variety of levels (i.e., from individual physiology to societal life–work balance). Furthermore, the term recognizes that sustained time pressure at high levels is potentially harmful (Kleiner, 2014). Indeed, intense time pressure can act as a stressor (Goode, 1960; Roxburgh, 2004; Szollos, 2009), which is detrimental to mental well-being (Zuzanek, 1998; Roxburgh, 2004). This notion has received attention in stress-related research (e.g., Type A behaviors) (Frei et al., 1999).

Szollos’ (Szollos, 2009) conceptualization of chronic time pressure is important because researchers have recently applied the construct to important real-world contexts, outlined implications and produced interventions. Work has embraced behavioral, cognitive and theoretical perspectives. For example, Cśugnet et al. (2019) reported that time pressure has an effect on risky street-crossing decision-making. Relatedly, Huang et al. (2018) considered the impact of time pressure on driver behavior. More generally, authors have evaluated the effect of temporal constraints on work life balance (Schöneck, 2018), well-being (Ryu, 2016), and lifestyle choices (e.g., eating habits, Hsu, 2015).

Conceptually, studies have investigated whether time pressure is stable across situations (home and work; Kleiner, 2014), cultures (Nordic countries; Gunnarsdottir et al., 2014), and genders (Tyrkkoe and Karlqvist, 2015). It is important to consider whether cultural and individual differences effect the perception, and consequences of time pressure because variations limit the usefulness of generalizations.

Additionally, researchers have investigated relationships between chronic time pressure and psychological well-being. A notable example is Gunnarsdottir et al. (2015), who found an association between parents’ subjective time pressure and increased mental health problems among children. Acknowledging that chronic time pressure is an unavoidable experience of daily life in industrialized societies, several studies have examined the effectiveness of alleviating strategies and therapeutic interventions. Examples include using teamwork (clarifying demands and setting priorities) to moderate the relationship between time pressure and exhaustion (Krause et al., 2017), mindfulness training (to counterbalance acceleration in social and working life and social relationships) (Kristensen, 2018), and downtime (a state of physical relaxation and psychological detachment) (Dugan and Barnes-Farrell, 2017).

### The Present Study

Researchers frequently use self-report measures (i.e., questionnaires and diaries) to assess feelings and perception of time pressure. Hence, scales represent an established, reliable and valid methodological tool for assessing the construct. Noting this, the present study designed a scale that incorporated the multidimensional aspects of CTP outlined by Szollos (2009). This was necessary for two reasons. Firstly, it facilitated psychometric evaluation of Szollos (2009) conceptualization of CTP. Particularly, analysis enabled the authors to determine whether measurement models provided adequate evidence for the existence of related, but discrete factors (time shortage proper and being rushed). At a general level, the study also offered insights into the dimensionality of CTP.

Secondly, previous self-report measures have been limited because there is no universal measure of CTP. Studies typically use either single questions (i.e., the General Social Survey), or small item groups, which assess only global (overall) perceptions of time pressure. Illustratively, the General Social Survey measures time pressure via a single item questioning how often the respondent feels rushed (Kleiner, 2014). Regarding small item groups, a typical example is Ackerman and Gross (2003) (i.e., “I feel a lot of time pressure in my life,” “I really feel the pressure of time passing in my life,” and “I am always in a hurry.”). A further often cited measure is Putrevu and Ratchford (1997). This comprises five-items related to shopping, which index: feeling pressed for time, being in a hurry, having limited/enough amount of time, and fastness. Accordingly, researchers frequently adapt these items for the purpose of their studies (e.g., Teng et al., 2010; Leischnig et al., 2011).

The current study represented the first step in the development of an established measure of CTP. This is an important development in time pressure research because it builds on Szollos (2009) review of literature and is conceptually congruent with his conclusions. Moreover, developing a robust measure of CTP facilitates context comparisons. This is important because the use of myriad measures conflates assessment of environment variations.

An additional objective was to examine convergent validity of the resultant measure. Specifically, this comprised a comparison with an established and relevant scale (the 10-item Perceived Stress Scale by Cohen and Williamson, 1988; PSS-10). Convergent validity is useful when designing a scale because it indicates the extent to which a measure aligns with a construct it should relate to according to theoretical predictions. Previous research consistently demonstrates that perceptions of stress correlate largely with time pressure. For example, Kourmousi et al. (2015) evidenced a correlation of 0.50 between time management pressures/issues among teachers (i.e., if a teacher can find the time to meet all significant professional or personal needs) and perceived stress. This study anticipated a similar pattern.

## Materials and Methods

### Participants

#### Study 1

A sample of 401 respondents (326 women, 81% and 74 men, 19%) participated in this study. The overall mean age was 26.20 (*SD* = 11.799, range 18–68 years). The mean age for men was 28.64 (*SD* = 12.488; range = 18–68 years), and the mean age for women was 25.66 (*SD* = 11.604; range = 18–67 years). Respondents included university students (24%) and employees from various occupational sectors (76%). Specifically, 21% from the educational sector; 13% public services and administration; 8% accountancy, banking and finance; 16% healthcare; 3% recruitment and HR; 6% retail and sales; 7% business and management. Recruitment was via social media, university staff email, and through local stakeholders (businesses and vocational classes). Involvement was voluntary and responses anonymized. Participants could withdraw up to 4 weeks after data collection. Exclusion criteria required that respondents were at least 18 years of age. Assessment of univariate skewness and kurtosis (Table 1) indicated no concerns, as values fell within the recommended range of −2.0 to +2.0 (Byrne, 2010). Conversely, multivariate non-normality existed, as Mardia’s (1970) kurtosis (*b2p* = 12.178, *p* < 0.001) and skewness (*b1p* = 13.980, *p* < 0.001) tests suggested significant deviation from normal distribution.

**TABLE 1:** Summary statistics for Study 1 and Study 2 items.

	**Study 1**				**Study 2**			
		
**CTPI item**	***M***	***SD***	**Skewness**	**Kurtosis**	***M***	***SD***	**Skewness**	**Kurtosis**
Q1	3.70	1.003	–0.741	0.047	3.66	0.971	–0.655	0.068
Q2	3.27	1.038	–0.315	–0.947	3.15	1.010	–0.240	–0.974
Q3	3.51	1.096	–0.469	–0.699	3.38	1.078	–0.179	–1.050
Q4	3.58	0.949	–0.388	–0.654	3.39	0.884	–0.246	–0.876
Q5	3.01	0.955	0.054	–1.034	2.84	0.895	0.268	–0.646
Q6	2.96	1.095	0.079	–1.153	2.93	1.037	0.069	–1.033
Q7	2.91	1.059	0.190	–0.927	2.74	1.069	0.255	–0.855
Q8	3.67	0.968	–0.698	–0.157	3.55	0.693	–0.590	–0.464
Q9	3.34	1.051	–0.297	–0.968	3.20	1.071	–0.125	–1.126
Q10	2.55	1.001	0.445	–0.622	2.65	1.003	0.341	–0.743
Q11	3.78	1.067	–0.802	–0.140	3.85	1.052	–0.849	–0.099
Q12	3.13	1.085	–0.083	–0.901	2.99	1.130	0.064	–0.925
Q13	3.86	1.113	–0.947	0.047	3.93	1.136	–1.081	0.390
Q14	3.30	0.982	–0.359	–0.731	3.07	0.937	–0.136	–0.665
Q15	2.97	1.014	0.056	–1.027	2.96	0.990	0.164	–1.016
**PSS-10 item**								
Q1	2.93	1.078	0.048	–0.627	2.96	1.102	0.086	–0.736
Q2	3.08	1.088	–0.005	–0.671	3.05	1.104	–0.014	–0.612
Q3	3.74	1.053	–0.468	–0.523	3.82	1.036	–0.716	0.055
Q4	2.66	0.966	0.300	–0.425	2.63	0.943	0.368	–0.560
Q5	2.87	0.867	0.033	–0.366	2.83	0.836	0.077	–0.449
Q6	3.02	1.024	0.216	–0.559	2.96	0.987	0.152	–0.516
Q7	2.78	0.876	0.022	–0.285	2.68	0.859	–0.044	–0.418
Q8	2.99	0.897	–0.001	–0.305	2.89	0.903	0.220	–0.357
Q9	3.04	1.073	–0.048	–0.692	2.95	1.082	–0.109	–0.798
Q10	2.76	1.181	0.245	–0.788	2.72	1.179	0.382	–0.657

*CTPI, Chronic Time Pressure Inventory; PSS-10, 10-item Perceived Stress Scale.*

#### Study 2

The Study 2 sample comprised 163 respondents (133 women, 82% and 30 men, 18%). Mean sample age was 19.15 (*SD* = 2.886, range = 18–36 years). For men, mean age was 20.21 (*SD* = 5.747, range = 18–36 years), and 18.92 for women (*SD* = 1.676, range = 18–29 years). Of the sample, 71% were university students and 29% were employees from different occupations. Specifically, 10% from the educational sector; 8% public services and administration; 7% healthcare; 4% business and management. The same recruitment procedure occurred as for Study 1. As with the Study 1 data, acceptable univariate skewness and kurtosis existed (i.e., all between −2.0 to +2.0) (Table 1). In addition, non-normality existed, as Mardia’s (1970) kurtosis (*b2p* = 5.351, *p* < 0.001) and skewness (*b1p* = 35.401, *p* < 0.001) inferred significant deviation.

### Measures

Study 1 and Study 2 both used the Chronic Time Pressure Inventory (newly devised by the study authors) and the Perceived Stress Scale (Cohen et al., 1983).

### The Chronic Time Pressure Inventory

An extensive review of literature on time pressure by the study authors occurred resulting in different but related operationalizations, for example ‘work demand’ (e.g., Boles and Adair, 2001), ‘time constraints’ (e.g., Cahir and Morris, 1991), ‘time urgency’ (e.g., Landy et al., 1991), ‘time pressure’ (e.g., Hamermesh and Lee, 2007). Notably (as alluded to in the Introduction), an absence of psychometrically validated measures existed, and in some instances single-item measures assessed time pressure (e.g., Hamermesh and Lee, 2007). Szollos’s (2009) review offered a thorough assessment of the status of time pressure literature and a plausible conceptualization of the construct. Thus, using this theoretical framework, the development of initial statements occurred. The study authors independently devised the statements to reflect core features of Szollos’s (2009) conceptualization. This resulted in 15 items following the removal of items indicative of duplication. A group of four experienced academics reviewed the measure, concluding that the scale appeared to assess features of time shortage, pressure, and feeling rushed. This procedure offered satisfactory face validity (e.g., see Macaskill and Taylor, 2010). The scale comprised 15 statements (e.g., “I always run out of time”) where participants are asked to rate each as it applies to them using a Likert response format from 1 (Strongly disagree) to 5 (Strongly agree).

### The Perceived Stress Scale (Cohen et al., 1983)

The Perceived Stress Scale, a global stress measure, assessed the extent that respondents perceive life to be unpredictable, uncontrollable, and overloading (Golden-Kreutz et al., 2004). The PSS detects existing stressful circumstances and background extraneous stressors. The present study used the 10-item version (PSS-10), which asks about thoughts and feelings over the last month. Items appear in the form of statements (e.g., “In the last month, how often have you felt nervous and stressed?”) and participants indicate their level of agreement on a 5-point Likert scale ranging from 0 (Never) to 4 (Very often). Summation of item scores produces an overall stress score; higher scores indicate greater levels of perceived stress. In addition, the scale possesses two underlying factors of ‘Distress’ and ‘Coping’ (Denovan et al., 2019). The PSS-10 possesses established psychometric properties (internal consistency, test-retest reliability and factor structure) (Cohen and Williamson, 1988; Denovan et al., 2019).

### Procedure

Prior to participation, potential respondents read the study brief. This contained background information about the nature of the study, and outlined the conditions and requirements of involvement. Only consenting participants progressed to the online measures hosted by Qualtrics. Further instructions asked participants to take their time, complete all questions, and answer questions openly/honestly. The online self-report measure comprised three subsections: demographic information (completed first), time pressure and perceived stress. To eliminate order effects, measure sequence rotated across respondents. This procedure was identical for Study 1 and Study 2.

### Ethics

The research team gained ethical authorization for the project (Evaluating the Chronic Time Pressure Inventory). The study investigated the development of a new measure of chronic time pressure in two independent samples. Following formal submission, the Director of the Research Institute for Health and Social Change and the Manchester Metropolitan University Faculty of Health, Psychology and Social Care Ethics Committee granted ethical approval.

### Analytic Strategy

Psychometric evaluation of the Chronic Time Pressure Inventory (CTPI) advanced through a number of sophisticated analytical procedures. These involved Horn’s parallel analysis, exploratory factor analysis (EFA using maximum likelihood), and confirmatory factor analysis (CFA). Parallel analysis (PA) determined the number of factors representing the CTPI. This is an empirically supported approach for establishing the quantity of factors underlying a measure (Pallant, 2007). PA involved random resampling of raw data (O’Connor, 2000). Eigenvalues higher than random data eigenvalues represented underlying factors. EFA (SPSS 25) with the recommended number of factors subsequently provided details of item loadings (Çokluk and Koçak, 2016).

Next, CFA (AMOS25) examined data-model fit. Given the presence of non-normality, CFA used ML estimation with bootstrapping (2000 resamples) to produce standard error estimates and confidence intervals (bias-corrected at the 95% confidence level) and *p*-values (Byrne, 2010). Naïve bootstrapping is a robust alternative to other ML robust approaches (e.g., Satorra–Bentler chi-square), and operates successfully even when data evidences extreme non-normality (Nevitt and Hancock, 2001).

Assessment of model fit included the chi-square statistic (χ*^2^*), Comparative Fit Index (CFI), Root-Mean-Square Error of Approximation, RMSEA, and Standardized Root-Mean-Square Residual, SRMR. RMSEA scrutiny involved reference to its 90% confidence interval (CI). Values > 0.90 imply good fit for CFI (Hopwood and Donnellan, 2010). Values of 0.05, 0.06–0.08, and 0.08–1.0 indicate good, satisfactory and marginal RMSEA and SRMR (Browne and Cudeck, 1993). Model comparison comprised Akaike’s Information Criterion (AIC). Lower values signify superior fit.

Internal consistency assessment followed guidelines recommended by Viladrich et al. (2017). This comprised the testing of equality constraints via SEM-based congeneric, tau equivalent and parallel models prior to estimating reliability coefficients (in this instance alpha and omega). The congeneric imposes the least restrictive assumptions, supposing that all scale items measure the same latent construct, with potentially different degrees of precision and error. The tau equivalent assumes that scale items measure the same latent construct with the same degrees of precision, but with potentially different error. The parallel imposes the most restrictive assumptions, supposing that items measure the same construct, with identical degrees of precision and error (Montero-Marin et al., 2014). Analysis considered chi-square differences among these models. However, because Study 1 involved a moderately large sample, consultation of fit indices less sensitive to sample size (CFI and SRMR) occurred, with 0.05 as an acceptable cut-off for changes in these (Little, 1997).

Cronbach’s alpha examined internal consistency of the CTPI following data-model inspection and testing of equality constraints within a single group. Coefficient omega (ω) also measured reliability (using the Omega program; Watkins, 2013) given this more successfully estimates reliability than alpha (Deng and Chan, 2017). Subsequently, convergent validity testing occurred. This included correlating CTPI with the PSS-10. Lastly, analysis involved inspecting associations between gender, age, CTPI and PSS-10. This project adopted Cohen’s (1988) criteria for judging the magnitude of associations. Specifically, 0.1–0.29 indicates a weak correlation; 0.3–0.49 suggests a moderate relationship; and 0.50 or greater infers a strong correlation.

The analysis procedure for Study 2 included (in an independent sample) replication and testing of the superior Study 1 model via CFA and assessment of alpha and omega reliability. Additionally, multi-group CFA evaluated invariance of the superior model by comparing Study 1 and Study 2 data at the configural (factor structure), metric (factor loadings), scalar (item intercepts), and residual (item residuals) levels. Assessment of fit at each stage involved consultation of Chen’s (2007) criteria (CFI difference ≤ 0.01 and RMSEA ≤ 0.015). As with Study 1, the final stages of analysis comprised a test of convergent validity (i.e., comparing CTPI with PSS-10) alongside exploring associations between gender, age, CTPI and PSS-10.

## Results

### Study 1

Parallel analysis using 1000 resamples indicated that two factors existed, given two factors evinced higher values compared with random data (factor 1 eigenvalue = 5.297 vs. 1.34; factor 2 eigenvalue = 1.533 vs. 1.265). EFA indicated a satisfactory item correlation matrix, Bartlett’s Test of Sphericity *p* < 0.001; and good sampling adequacy, Kaiser-Meyer-Olkin = 0.900. The factors accounted for 45.534% of variance. Consideration of factor loadings revealed that item 14 (‘I am not concerned about being late and missing things’) loaded poorly (0.212). In addition, item 7 (‘I am so busy I mess things up’) evidenced cross loading (factor 1 loading = 0.389; factor 2 loading = 0.329). EFA following removal of these items explained 48.792% of the variance. All items loaded above the minimum threshold of 0.32 (Tabachnick and Fidell, 2014) and, apart from item 6 (‘I feel in control of how I spend my time’, loading of 0.387), exceeded 0.4 (Norman and Streiner, 1994).

Five items loaded on Factor 1, and eight items loaded on Factor 2 (Table 2). Items belonging to Factor 1 (labeled as ‘Feeling Harried’) referred to a negative sense of feeling rushed to an extent where time concerns generate anxiety and worry. Items informing Factor 2 (named ‘Cognitive Awareness of Time Shortage’) referred to a cognizance of not having enough time to complete tasks, to do the things they enjoy (see Appendix for final scale).

**TABLE 2:** EFA and CFA factor loadings for Study 1 and Study 2.

	**Study 1**								**Study 2**		
		
	**EFA factors**		**CFA factors^a^**						**CFA factors^a^**		
			
			**One-factor**	**Two-factor**		**Bifactor**			**Bifactor**		
						
**CTPI item**	**1**	**2**	**1**	**1**	**2**	**1**	**2**	**3**	**1**	**2**	**3**
Q4	0.511		0.673^∗∗^	0.681^∗∗^		0.001		0.723^∗∗^	0.180		0.481^∗∗^
Q8	0.659		0.479^∗∗^	0.549^∗∗^		−0.348		0.459^∗∗^	0.685		0.545^∗∗^
Q10	0.685		0.570^∗∗^	0.632^∗∗^		−0.170		0.600^∗∗^	−0.069		0.703^∗∗^
Q11	0.853		0.584^∗∗^	0.676^∗∗^		−0.638		0.564^∗∗^	0.152		0.563^∗∗^
Q12	0.645		0.703^∗∗^	0.757^∗∗^		−0.193		0.718^∗∗^	−0.256		0.768^∗∗^
Q1		0.592	0.450^∗∗^		0.508^∗∗^		0.444^∗^	0.339^∗∗^		0.411^∗^	0.373^∗∗^
Q2		0.553	0.445^∗∗^		0.508^∗∗^		0.475^∗^	0.317^∗∗^		0.372^∗^	0.251^∗^
Q3		0.555	0.610^∗∗^		0.648^∗∗^		0.345^∗∗^	0.539^∗∗^		0.309^∗^	0.512^∗∗^
Q5		0.661	0.484^∗∗^		0.539^∗∗^		0.369^∗∗^	0.408^∗∗^		0.403^∗^	0.483^∗∗^
Q6		0.387	0.615^∗∗^		0.631^∗∗^		0.312^∗^	0.537^∗∗^		0.060	0.557^∗∗^
Q7		0.629	0.378^∗∗^		0.455^∗∗^		0.498^∗∗^	0.243^∗∗^		0.470^∗∗^	0.282^∗^
Q9		0.490	0.578^∗∗^		0.616^∗∗^		0.365^∗^	0.491^∗∗^		0.287^∗^	0.397^∗∗^
Q13		0.449	0.680^∗∗^		0.672^∗∗^		0.207^∗∗^	0.660^∗∗^		0.389^∗^	0.558^∗∗^

CTPI, Chronic Time Pressure Inventory. ^a*^p < 0.05; ^∗∗^*p* < 0.001 (using bootstrap significance estimates).

**FIGURE 1:**
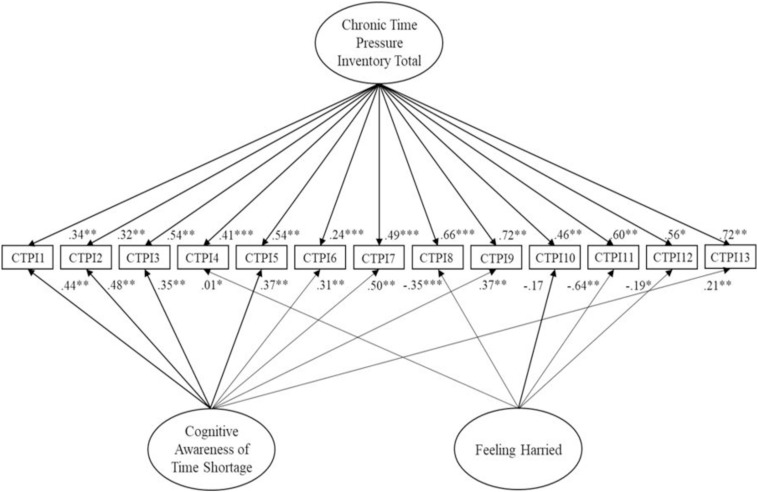
Two-factor bifactor model of the Chronic Time pressure Inventory for Study 1. Latent variables are represented by ellipses; measured variables are represented by rectangles; error is not shown but was specified for all variables. ^∗^*p* < 0.05; ^∗∗^*p* < 0.01; ^∗∗∗^*p* < 0.001 (using bootstrap significance estimates).

Analysis of a correlated two-factor model resembling the EFA results reported satisfactory fit overall (Table 3). All factor loadings (Table 2) exceeded 0.4 (Norman and Streiner, 1994), with 8 of the 13 items (62%) loading above the strict requirement of 0.6 by Hair et al. (1998). Further examination revealed that the subfactors correlated highly (0.747), suggesting conceptual overlap. Therefore, a bifactor model tested the notion of multidimensionality. Findings (Figure 1) indicated good fit overall.

**TABLE 3:** Fit indices for measurement and invariance models of the CTPI.

**Model**	**χ^2^**	***df***	**CFI**	**SRMR**	**RMSEA (90% CI)**	**AIC**
**Study 1 (*N* = 401)**						
One-factor	313.609^∗∗^	65	0.834	0.072	0.100 (0.087–0.109)	391.609
Two-factor	208.053^∗∗^	64	0.904	0.058	0.075 (0.064–0.087)	288.053
Bifactor	125.672^∗∗^	52	0.951	0.038	0.060 (0.046–0.073)	229.672
Reliability						
Congeneric	125.672^∗∗^	52	0.951	0.038	0.060 (0.046–0.073)	
Tau equivalent	212.846^∗∗^	75	0.907	0.067	0.068 (0.058–0.079)	
Parallel	244.249^∗∗^	86	0.894	0.074	0.068 (0.058–0.078)	
**Study 2 (*N* = 163)**						
Bifactor	84.972^∗∗^	52	0.935	0.056	0.063 (0.037–0.086)	
**Invariance (*N* = 564)**						
Configural	216.500^∗∗^	106	0.945	0.045	0.043 (0.035–0.051)	
Metric	257.485^∗∗^	128	0.935	0.045	0.042 (0.035–0.050)	
Scalar	282.369^∗∗^	141	0.929	0.046	0.042 (0.035–0.049)	
Residual	302.872^∗∗^	157	0.927	0.047	0.041 (0.034–0.048)	

*^∗∗^Indicates *p* < 0.001.*

All factor loadings (apart from item 6, loading of 0.243) exceeded 0.32 on the general factor. In comparison, some of the items pertaining to Factor 1 did not load significantly, suggesting that these more directly inform a general factor. Furthermore, negative item loadings existed on Factor 1, which can happen unexpectedly in bifactor solutions (e.g., Chen et al., 2012) due to a crossover suppression effect (Paulhus et al., 2004). All items (apart from 6 and 13) loaded above 0.32 on Factor 2. However, direct comparison of factor loadings and observation of the average weights (specifically, Factor 1 = −0.269; Factor 2 = 0.376; Factor 3 = 0.507) suggested that, overall, items loaded more highly on a general factor. Discrete variance does appear to exist, particularly in the case of Factor 2.

Lastly, CFA analysis considered a unidimensional model (as a null test of whether a single factor explains sufficient variance; Drinkwater et al., 2017). Unsatisfactory fit existed on all criteria but SRMR (Table 3). This indicated that a single factor solution did not represent a good fit to the data. In addition to better data-model fit across indices, AIC supported superior fit of the bifactor model, given this was lower (229.672) than the two-factor (288.053) and unidimensional (391.609) solutions.

Table 3 demonstrates fit of the reliability models for the bifactor solution. The congeneric model fitted the best. This is unsurprising, however, given this is the least restrictive and avoids assumptions about constant means and variances (Dunn et al., 2014). The tau equivalent and congeneric model exhibited a significant chi-square difference, χ^2^ (*df* = 23) = 87.174, *p* < 0.001, yet CFI and SRMR differences less than 0.05 existed. Similarly, a significant chi-square difference occurred for the parallel and tau equivalent models, χ^2^ (*df* = 11) = 31.403, *p* < 0.001, but analysis evidenced CFI and SRMR differences below recommended cut-offs. To exercise caution, however, analysis considered alpha as a lower bound estimate of the CTPI’s reliability, with omega representing a more accurate model-based interpretation of scale reliability (Chen et al., 2012).

Alpha was good for the total scale (α = 0.854), Feeling Harried (FH) (α = 0.795), and Cognitive Awareness of Time Shortage (CA) (α = 0.800). Coefficient omega conveyed similar (yet slightly higher) outcomes: good reliability for a general factor (ω = 0.878), for FA (ω = 0.819), and for CA (ω = 0.809). Omega hierarchical was high for a general CTPI factor (ω*h* = 0.702); however, lower estimates existed for FH (ω*h* = 0.133) and CA (ω*h* = 0.341). Common variance (ECV) exhibited comparable results, as total CTPI accounted for 66.9% whereas FH and CA explained 11 and 22.1% respectively. The percentage of uncontaminated correlations (PUC) was 51.3%. Reise et al. (2013) advise that if PUC < 0.80 and ECV > 0.60 and ωh > 0.70, then a scale can be interpreted as largely unidimensional.

A test of convergent validity with the PSS-10 reported large correlations between total CTPI with total PSS-10, PSS-10 Distress, and PSS-10 Coping factors (Table 4). Similarly, FH evidenced large correlations with total PSS-10, PSS-10 Distress, and PSS-10 Coping. CA demonstrated moderate correlations with the PSS-10 outcomes. Examining associations between age and gender with CTPI, CTPI subfactors, PSS-10, and PSS-10 subfactors indicated significant (albeit small) positive correlations between gender, total PSS-10 and PSS-10 Distress. Further inspection revealed that females reported higher average levels of total PSS and PSS-10 Distress (Males: total PSS = 28.337, PSS-10 Distress = 17.297; Females: total PSS = 30.217, PSS-10 Distress = 18.862). This finding is consistent with previous studies investigating the PSS-10 (see Denovan et al., 2019). In addition, females indexed greater means of CTPI, FH and CA than males (though this was non-significant). FH and all PSS-10 variables demonstrated small negative correlations with age (Table 4).

**TABLE 4:** Intercorrelations among total CTPI, CTPI subscales, total PSS-10, PSS-10 subscales, gender and age for Study 1 and Study 2.

**Variable**	***M***	***SD***	**1**	**2**	**3**	**4**	**5**	**6**	**7**	**8**
**Study 1**										
(1) Total CTPI	43.107	8.071		0.847^∗∗^	0.910^∗∗^	0.600^∗∗^	0.599^∗∗^	0.500^∗∗^	0.079	0.030
(2) Feeling Harried	16.281	4.00			0.552^∗∗^	0.596 ^∗∗^	0.588^∗∗^	0.508^∗∗^	0.088	0.122^∗^
(3) Cognitive Awareness of Time Shortage	26.825	5.138				0.479^∗∗^	0.482^∗∗^	0.390^∗∗^	0.046	–0.097
(4) Total PSS-10	29.870	7.418					0.967^∗∗^	0.888^∗∗^	0.098^∗^	−0.169^∗^
(5) Distress	18.573	5.074						0.741^∗∗^	0.120^∗^	–0.191^∗∗^
(6) Coping	11.296	2.829							0.043	−0.101^∗^
(7) Gender	1.820	0.388								−0.098^∗^
(8) Age	26.200	11.799								
**Study 2**										
(1) Total CTPI	42.690	7.673		0.856^∗∗^	0.926^∗∗^	0.585^∗∗^	0.576^∗∗^	0.475^∗∗^	0.131	0.024
(2) Feeling Harried	16.831	3.607			0.599^∗∗^	0.533^∗∗^	0.507^∗∗^	0.469^∗∗^	0.131	0.070
(3) Cognitive Awareness of Time Shortage	25.858	4.948				0.518^∗∗^	0.524^∗∗^	0.396^∗∗^	0.099	–0.045
(4) Total PSS-10	31.089	7.079					0.962^∗∗^	0.858^∗∗^	0.174^∗^	–0.021
(5) Distress	19.438	4.992						0.685^∗∗^	0.207^∗^	–0.013
(6) Coping	11.651	2.654							0.076	–0.033
(7) Gender	1.820	0.389								−0.165^∗^
(8) Age	19.150	2.886								

*^∗^Indicates *p* < 0.05; ^∗∗^indicates *p* < 0.001.*

### Study 2

**FIGURE 2:**
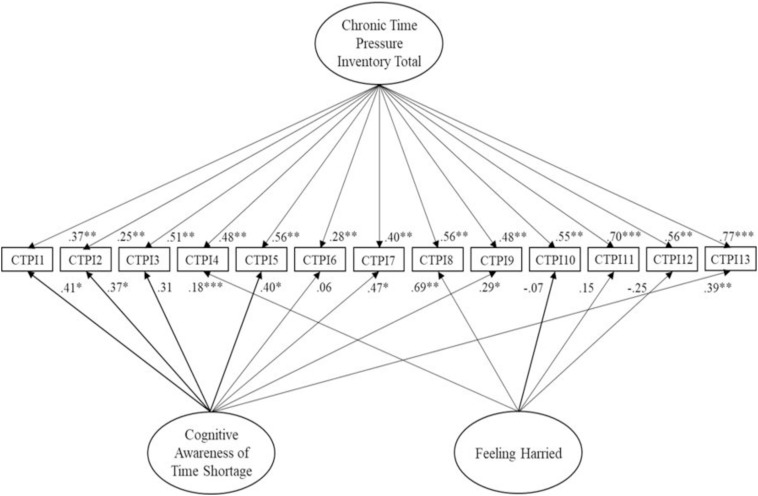
Replication of the bifactor model of the Chronic Time pressure Inventory for Study 2. Latent variables are represented by ellipses; measured variables are represented by rectangles; error is not shown but was specified for all variables. ^∗^*p* < 0.05; ^∗∗^*p* < 0.01; ^∗∗∗^*p* < 0.001 (using bootstrap significance estimates).

Replication of the superior bifactor solution from Study 1 (Figure 2) resulted in satisfactory to good fit. Factor loadings (Table 2) followed a similar pattern to Study 1. Specifically, direct comparison across factors and observation of average weights (Factor 1 = 0.138; Factor 2 = 0.337; Factor 3 = 0.497) suggested that a general factor possessed higher factor loadings overall. In addition, Factor 2 accounted for a reasonable quantity of discrete variance.

Multi-group analysis comparing Study 1 and Study 2 reported good model fit at the configural stage (Table 3). At the metric level, a satisfactory CFI difference of 0.01 existed alongside an RMSEA difference of 0.001. Testing scalar invariance reported acceptable CFI (0.006) and no change in RMSEA. At the residual invariance level, satisfactory CFI and RSMEA differences existed (0.002 and 0.001 respectively). Findings support invariance of factor structure, loadings, intercepts and residuals.

Consistent with Study 1, alpha reliability was good for the total scale (α = 0.858), FH (α = 0.820), and CA (α = 0.759). Coefficient omega additionally indicated good reliability for these components (general factor ω = 0.863; FH ω = 0.799; CA ω = 0.778). For a general Chronic Time Pressure factor, omega hierarchical was high (ω*h* = 0.728); yet lower for FH (ω*h* = 0.039) and CA (ω*h* = 0.299). In terms of ECV, total CTPI explained 68.3%, and FH and CA accounted for 11.7% and 20.1%. PUC was identical to Study 1 (51.3%). These results support reliability outcomes from Study 1, and suggest that the CTPI is principally unidimensional according to Reise et al. (2013).

Convergent validity analysis for Study 2 (i.e., compared with the PSS-10) demonstrated large correlations between total CTPI with total PSS-10 and PSS-10 Distress, and a moderate association with PSS-10 Coping (Table 4). FH and CA also evinced large associations with total PSS-10 and PSS-10 Distress, and a moderate correlation with PSS-10 Coping. Consistent with Study 1, gender evidenced small (yet significant) positive correlations with total PSS-10 and PSS-10 Distress only. Females reported higher mean levels of total PSS and PSS-10 Distress (Males: total PSS = 28.498, PSS-10 Distress = 17.270; Females: total PSS = 31.674, PSS-10 Distress = 19.927). Females indexed greater averages of CTPI, FH and CA than males (though non-significant). Age, however, did not demonstrate any significant associations with CTPI or PSS-10 variables (Table 4).

## Discussion

Psychometric evaluation of the Chronic Time Pressure Inventory (CTPI) revealed that the scale reflected a general dimension comprising two discrete, but overlapping temporal factors: Cognitive Awareness of Time Shortage (CA) and Feeling Harried (FH). Consistent with Szollos (2009), CA and FA indexed negative features associated with the subjective experience of time shortage. These specifically referenced worry, feeling rushed and a sense of pressure. Congruent with this notion, analysis found positive correlations in the medium to large range between CTPI measures (overall and factors) and perceived stress. Concomitantly, items centered thematically on lack of control and the inability to schedule and complete tasks. This conceptualization is consistent with preceding research, which has reported an association between lack of apparent control and the negative effects of time pressures (Teuchmann et al., 1999). Indeed, feelings of control can generally decrease the negative effects of time pressure, although this relationship varies as a function of situation (i.e., workload) and individual differences (i.e., level of neuroticism) (Teuchmann et al., 1999).

The emergent factors CA and FH shared features with the notions of ‘time shortage’ (perceived lack of time), and ‘being rushed’ (sense of time passing quickly) outlined by Szollos (2009). Although, FH and CA correlated positively (these factors shared between 30 and 36% variance) there was considerable theoretical divergence. Thus, despite possessing two items referencing ‘hurry’ and ‘pressure,’ CA aligned with time shortage. Explicitly, CA comprised items that were generally synonymous with time management issues. Specifically, reflected judgments about perceived absence of time, especially insufficiency (e.g., “There aren’t enough hours in the day”). Thus, the emphasis with CA was objective awareness of time shortage rather than negative, affective consequences of time pressure.

Contrastingly, FH more closely aligned to Szollo’s delimitation of feeling rushed. Particularly, FH items indexed subjective feelings of apprehension, worry, anxiety and frustration arising from the experience of the perceived rapid passage of time (e.g., “The days fly by without me ever getting anything done” and “I feel rushed to do the things that I have do”).

Although these observations are intuitively consistent, it is important to interpret them cautiously because the CTPI possessed a complex structure, which best fitted a bifactor solution. This indicated the presence of a latent structure where items loaded on a general factor indexing negative features associated with the subjective experience of time shortage. In practice, findings recommend the use of total scores rather than independent subscales when assessing general population samples as the CTPI is, for the most part, unidimensional. A degree of non-redundant variance existed, however, particularly for CA. Thus, the subscales can be used when administering the scale, but in the company of general scores. This inference accords with existing research concerning bifactor solutions (e.g., McElroy et al., 2018). In addition, omega reliability indicated that the CTPI was reliable and that a general factor accounted for a sufficient proportion of variance in scale scores. Therefore, in terms of administering the CTPI in practice, the authors recommend summing the values of the Likert scale to form composite scores to represent levels of chronic time pressure, with the subscales usable for information purposes.

Furthermore, although CA in particular possessed unique variance the subfactors were orthogonal and accordingly demonstrated conceptual overlap. The overall analysis was compatible with the notion that the concept of time pressure is an overarching designation that subsumes terms related to time shortage and being rushed. Additionally, the CTPI demonstrated invariance at the strictest level across the two studies. This indicated that the scale measured the same construct across the two studies without any notable measurement bias (González-Blanch et al., 2018). While this study supported the presence of factors that corresponded with Szollos (2009) conceptualization and evidenced factorial validity, further research is required to establish fully the legitimacy of the proposed theoretical distinction. Ensuing work on CTPI needs also to assess the temporal stability of the measure to ensure that it possesses test–retest reliability.

Supplementary evidence supporting the psychometric robustness of the CTPI was apparent. Explicitly, the measure demonstrated content-related validity. Concerning face validity, scrutiny of the items by an academic panel ensured that the CTPI accurately assessed core elements of perceived time shortage (see Macaskill and Taylor, 2010). Additionally, CTPI analyses indicated convergent validity; the CPTI strongly positively correlated with the PSS-10. The observed relationship was similar to those noted in other related studies (e.g., Ackerman and Gross, 2003, time pressure and stress; Kourmousi et al., 2015, time management pressures/issues and perceived stress).

Following studies should seek to establish concurrent validity by comparing the performance of the CPTI alongside similar extant measures. Although, myriad studies investigated time pressure these have used a range of instruments. Hence, consideration of the CTPI alongside a subset of these would usefully help to establish the scale’s psychometric credibility. In this context, studies could examine CTPI performance alongside the Time Pressure Scale (Roxburgh, 2004) and the single-item measure used by Hamermesh and Lee (2007) (i.e., “How often do you feel rushed or pressed for time?”). Another related extension to the present paper could examine the extent to which CTPI predicted scores on time pressure-related variables, e.g., “Lack of efficiency” and “Forced to cut down on lunch time” (cf. Tyrkkoe and Karlqvist, 2015).

### Limitations and Future Research

Several limitations existed. Notably, although the studies utilized samples comprising a range of occupations, both samples were predominantly female. In addition, the age range was rather restrictive with relatively youthful mean ages and the majority of participants being under the age of 35 (80% for Study 1; 98% for Study 2). Study 2 sample also contained a preponderance of university students (71%). These features limit the generalizability of the results to samples of various ages and replication is required with populations that are more heterogeneous. A second limitation relates to use of self-report data, which is associated with recognized limitations including response bias (Denovan et al., 2019). Including supplementary assessment methods (e.g., physiological assessment), when measuring chronic time pressure would be useful in future. Lastly, as aforementioned analysis did not include test-retest reliability.

The present study did adopt, nonetheless, certain strategies to circumvent typical issues inherent with the use of a single time point (a cross-sectional design). Specifically, this approach is frequently criticized because it can result in common method variance (CMV) and the inability to draw causal conclusions (Spector, 2019). The present study minimized the possibility of CMV by using procedural remedies (Krishnaveni and Deepa, 2013). Importantly, the researchers created methodological separation between the CTPI and the PSS-10. Particularly, the authors made it clear within the participant instructions that the measures assessed different constructs. Furthermore, the two measures used dissimilar response scales. These factors created psychological distance between the CTPI and the PSS-10 (Podsakoff et al., 2003).

Finally, the researchers reduced the likelihood of evaluation apprehension and social desirability effects by providing instructions that emphasized that there were no were no right or wrong answers and that respondents should answer questions as honestly as possible. The notion of causality was not important in the context of the present study, as assessment of the CTPI against a criterion (PSS-10) was correlational in nature and intended to assess convergence. Relatedly, the strength of correlations suggested that the scale was not simply indexing stress, but captured additional variance.

Accordingly, subsequent research may wish to examine the extent to which time pressure causes stress. This is important because significant previous research acknowledges that, whilst stress and time pressure result from an interaction between the individual and the environment, there are circumstances where the assumption is that time pressure causes ensuing stress. This is certainly true within medical literature, which attributes high stress and contemporary stress-related conditions (e.g., hypertension) to time shortage/pressure (Dossey, 1982). A way to determine the direction of the chronic time pressure-stress relationship is to conduct a longitudinal study with multiple intervals. This approach, combined with sophisticated analytical techniques such as latent growth curve modeling, would determine how chronic time pressure and stress change (or remain stable) as a function of time. The outcome would inform the development of interventions designed to alleviate the negative effects of these factors.

Relatedly, future research should consider also the degree to which perception of chronic time pressure varies as a function of context (i.e., work vs. home life). It may be that the situational factors within occupational settings, such as deadlines, targets and organizational level may exacerbate feeling of time pressure and accordingly have a more negative effect on the individual. Alongside this, studies could evaluate the mediating/moderating influence of individual differences. Illustratively, work historically has demonstrated that Type A personality (hard driving, persistent, involved in work) is associated with stress-related illness (Caplan and Jones, 1975). From this perspective, an evaluation of the effects of time management training may also prove informative.

In terms of applications, the CTPI provides a solid platform for further empirical investigation of time pressure at an individual and societal level. Explicitly, it provides an expedient measure, which researchers can use in myriad contexts (occupational, educational, health, etc.) for complementing understanding of stress, wellbeing, life satisfaction and work-life balance.

In terms of implications, the CTPI provides a solid platform for further empirical investigation of time pressure at an individual and societal level. Explicitly, it provides an expedient measure, which researchers can use in myriad contexts (occupational, educational, health, etc.) for complementing understanding of stress, wellbeing, life satisfaction and work-life balance.

Specifically, the measure will assist researchers to identify groups that are vulnerable to chronic time pressure. This information will usefully inform policymaking and facilitate the design and implementation of appropriate health policies and interventions. This is important because preceding research has found that time pressure varies as a function of role. For example, Otterbach et al. (2016) reported that managing multiple roles (i.e., child rearing, income-earner, and a caregiver) was a cause of time pressure in a sample of Australian women born between 1973 and 1978. This finding accorded with Kleiner (2014), who noted that time pressure spanned contextual boundaries (e.g., home and at work). In this instance, strategies to ameliorate the negative effects of time pressure should focus on balancing diverse demands. This could involve identifying key stress points/times and making best use of available resources (i.e., health policies, family friendly leave and child-care policies) (Otterbach et al., 2016).

Using the CTPI to identify groups experiencing chronic time pressure recognizes the subjective nature of time pressure and the fact that health risks vary across contexts. Illustratively, time pressure resulting from professional activities (i.e., time constraints, challenges and uncertainties) produced negative emotions in hospital-in-the-home nurses that led them to take more risks on the road (Cśugnet et al., 2016). Furthermore, development of occupation specific coping strategies is important because rigid and inflexible coping patterns can exacerbate the risk of stress and health disorders (Krause et al., 2017).

## Data Availability Statement

The datasets generated for this study are available on request to the corresponding author.

## Ethics Statement

The studies involving human participants were reviewed and approved by The Manchester Metropolitan University Faculty of Health, Psychology and Social Care Ethics Committee. The patients/participants provided their written informed consent to participate in this study.

## Author Contributions

AD: questionnaire development, theoretical focus, analysis and write-up. ND: questionnaire development, theoretical focus, and write-up.

## Conflict of Interest

The authors declare that the research was conducted in the absence of any commercial or financial relationships that could be construed as a potential conflict of interest.

## Appendix

**Table:** Chronic Time Pressure Inventory. Instructions: Here are a number of statements that focus on whether you feel you have enough time in your life to do the things you want to do. For each statement, select the response (from 1 “*Strongly disagree*” to 5 “*Strongly agree*”) that most accurately captures your thoughts or feelings.

	**Strongly disagree (1)**	**Disagree (2)**	**Neither disagree nor agree (3)**	**Agree (4)**	**Strongly agree (5)**
(1) There aren’t enough hours in the day					
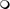
(2) I have enough time to do the things that I want to do (R)					
(3) I feel pressured to fit everything in					
(4) The days fly by without me ever getting everything done					
(5) I am often in a hurry					
(6) I feel in control of how I spend my time (R)					
(7) I should have more free time to do the things I enjoy					
(8) I worry about how well I use my time					
(9) I have enough time to properly prepare for things (R)					
(10) I think I won’t finish work that I set out to do					
(11) I feel disappointed with how I spend my time					
(12) I always run out of time					
(13) I feel rushed to do the things that I have to do					

*Summing all the items produces a total score. Items 4, 8, 10, 11, and 12 belong to ‘Feeling Harried’ (Factor 1); items 1, 2, 3, 5, 6, 7, 9, and 13 comprise ‘Cognitive Awareness of Time Shortage’ (Factor 2). Items with (R) need to be reverse-scored prior to analysis.*
